# Gendered differences in the prevalence and associated factors of dementia in Ghana: a cross-sectional survey

**DOI:** 10.1186/s12888-024-05856-3

**Published:** 2024-05-27

**Authors:** Precious Adade Duodu, Nutifafa Eugene Yaw Dey, Joshua Okyere, Bibha Simkhada, Caroline Barker, Warren Gillibrand, Padam Simkhada

**Affiliations:** 1 Department of Nursing, School of Human and Health Sciences, University of Huddersfield, Queensgate, Huddersfield, England, UK; 2 Department of Psychology, University of Ghana, Legon-Accra, Ghana; 3 Department of Population and Health, University of Cape Coast, Cape Coast, Ghana; 4 School of Nursing and Midwifery, College of Health Sciences, Kwame Nkrumah University of Science and Technology, Kumasi, Ghana; 5 School of Human and Health Sciences, University of Huddersfield, Queensgate, Huddersfield, England, UK

**Keywords:** Dementia, Gender, Ageing, Cross-sectional survey

## Abstract

**Background:**

Dementia as a global phenomenon has received significant attention in research due to the adverse effects it has on the daily functioning of its victims. Despite studies conducted in relation to the prevalence and associated factors of dementia in Ghana, not much attention has been paid to the influence of gender. The study, therefore, focused on estimating gender differences in the prevalence and associated factors of dementia in the Ashanti Region of Ghana.

**Methods:**

This study adopted a cross-sectional design with surveys to recruit 800 participants who were 45 years or older. The data was obtained using the standardized Rowland Universal Dementia Assessment Scale (RUDAS) together with information on the various associated factors. A series of logistic models comprising of the total sample model, male sample model, and female sample model were estimated to analyse the data. All data analyses were completed in Stata version 14.

**Results:**

The overall prevalence of dementia was 23.38% [95% CI:20.44, 26.31]. More females 24.56% [95% CI:20.81, 28.31] compared to males 21.31% [95% CI:16.57, 26.04] were at risk of dementia. Younger age, attaining formal education, and belonging to richer households were negatively associated with the risk of dementia. In the total sample model, younger age and attaining formal education were negatively associated with dementia risk. In the male-female stratified models, education and household wealth index were negatively associated with dementia risk in the male sample while age and education were negatively related to dementia risk in the female sample.

**Conclusion:**

The study concludes that there are gendered differences in the prevalence and factors associated with the risk of dementia in Ghana. As such, interventions and programmes to identify dementia cases must be gender sensitive. Specifically, when addressing dementia risk in males, interventions should be directed towards those with lower wealth status. Likewise, when developing programmes to mitigate dementia risk in women, particular attention should be given to women in the oldest age category.

**Supplementary Information:**

The online version contains supplementary material available at 10.1186/s12888-024-05856-3.

## Background

Over the past three decades, there has been a significant shift in the causes of mortality, with non-communicable diseases [NCDs] including dementia accounting for a higher burden of deaths and frailty. Dementia, an NCD, is “a syndrome, usually of a chronic or progressive nature, in which there is deterioration in cognitive function, accompanied by a decline in emotional control, social behaviour, or motivation” [1]. Approximately 57.4 million individuals globally are currently living with dementia or some form of cognitive impairments, and projections suggest that this figure will surge to 152.8 million by the year 2050 [2]. Nichols et al. [2] further report that sub-Saharan Africa (SSA) has one of the largest percentage changes in terms of projected dementia cases (357%). This situation makes dementia and cognitive impairments a serious public health concern in SSA. In Ghana, there is little empirical evidence on the spread and magnitude of the condition. One study [3] estimated the overall prevalence to be 5% among the general Ghanaian population. However, Nyame et al.’s [3] study was limited to only persons aged 70 years or older. Given that dementia can also affect individuals below the age of 70, the exclusion of younger population in Nyame et al.’s study [3] results in an incomplete understanding of the overall prevalence and patterns of dementia in Ghana. It is, therefore, imperative to understand the prevalence of dementia (cognitive impairment) and its associated factors within the Ghanaian context.

Given the adverse impact of dementia and cognitive impairment on the quality of life of the individual, the scientific community has been interested in understanding the associated factors in order to champion preventive measure, early detection and facilitate effective management. Evidence suggests that factors such as gender [2], socio-economic status [4], ageing [5], and lifestyle factors (i.e., level of physical activity, dietary practices, etc.) [6] are significantly associated with the risk of dementia. For instance, Nichols et al. [2]’s estimation of the global prevalence of dementia revealed that there were more women living with dementia than men; thus, suggesting a higher risk of dementia among females compared to males. Wang et al. [4]’s systematic review and meta-analysis have also reported that individuals in lower socio-economic status are more likely to be at risk of dementia.

There are several studies that have examined the factors associated with dementia risk [5–8]. For instance, Chaaya et al. [7] in a cross-sectional study reported that increasing age, perceived low income and having uncontrolled hypertension were associated with higher risk of dementia. Yet, the authors failed to report whether these associations differ significantly for males and females. Also, while Bich et al. [8] found the risk of dementia to be high among females, they do not show how significant predictors (i.e., low educational level, ageing, physical inactivity, and previous stroke) differs by gender. And so, it is clear that the existing body of literature on the associated factors of dementia does not account for variations in the risk by gender. This is a significant limitation in what is currently known about the factors associated with dementia risk. Hence, this study contributes to knowledge by providing evidence of gendered differences in the prevalence and associated factors of dementia risk. Men and women may face distinct challenges and risk factors. As such, gaining knowledge about gender-specific differences in dementia can inform the development of targeted screening and diagnostic strategies. This is essential for identifying individuals at risk and facilitating early intervention, potentially slowing down the progression of the disease. Moreover, tailoring diagnostic tools to account for gender-specific factors can improve the accuracy and timeliness of dementia diagnoses. Also, understanding gender-specific variations would inform the allocation of resources to areas where the prevalence of dementia may differ between men and women. Against this background, we investigated gender-specific differences in the prevalence and factors associated with dementia in the Ashanti Region of Ghana.

## Methods

### Research design and study setting

This study adopted a cross-sectional research design. Currently, Ghana does not have a national data that shows the regional disparities in terms of hotspots for dementia. This implies that all older people in Ghana have an equal chance to developing cognitive impairments. As such, selecting a study site required us to list all 16 regions in no specific order. Using Microsoft Excel’s random number generation tool, we selected the Ashanti region. The process was repeated for the districts until the final study sites were selected. To ensure representativeness, we randomly selected a mix of healthcare facilities including a university hospital, district hospitals, and a private healthcare facility in the Ashanti Region, Ghana. These facilities were the Ejisu Government Hospital, Juaben Government Hospital, University Hospital - Kwame Nkrumah University of Science and Technology (KNUST), Kumasi South Hospital, Manhyia District Hospital, Onwe Government Hospital; Kumasi Cheshire Home (a private healthcare facility), and Tafo Government Hospital. We expected that all the selected facilities would have a psychiatry or mental health unit. These facilities were selected at random by first listing all district, university and private hospitals in Microsoft Excel. This listing was done in no particular order to ensure randomness. We then relied on the random number generation tool in Microsoft Excel to generate 8 random numbers. Health facilities that whose numbers appeared in the randomly generated numbers were selected as the study sites. We have attached the capacity and breakdown of the healthcare facilities as a Supplementary File (see supplementary file 1).

### Sampling and sample size

As a study that follows a quantitative approach, randomisation was key for us to be able to generalise the findings. Therefore, probability sampling techniques were used to sample the target population. Specifically, simple random sampling was applied to recruit all of the study participants. To identify eligible participants, we collaborated closely with the administrative staff and healthcare providers at each facility. Upon arrival at the OPD, individuals meeting the age criteria were identified through their registration information or directly by healthcare staff during triage. These individuals were then approached by trained research personnel who explained the purpose and procedures of the study.

Regarding the apportionment of the sample at each facility, we followed a systematic approach to ensure representation while considering the varying attendee populations. For instance, 112 participants were randomly selected from each facility; however, at Kumasi Chesire Home where the attendee numbers were lower, we sampled a smaller proportion of participants (16) to maintain representativeness.

We calculated the sample size using the formula: $n\hspace{0.17em}=\hspace{0.17em}{Z}^{2*}{P}^{*}(1-P)/{E}^{2}$.

where:

*n* is the required sample size,

*Z* is the Z-score corresponding to the desired confidence level (e.g., for a 95% confidence level, = 1.96*Z* = 1.96),

*P* is the anticipated prevalence of the condition (expressed as a proportion, so 10% becomes 0.10),

*E* is the margin of error (0.09).

An earlier study conducted by Agyeman [9] in Ghana estimated the prevalence to be 6.6 for all ages. Considering the time lapse since Agyeman’s study [9], we anticipated the possibility of changes in dementia prevalence due to various factors such as population aging, evolving diagnostic criteria, and improved awareness leading to better detection rates. Hence, we cautiously adjusted our anticipated prevalence to 10% to account for potential increases over time. This resulted in an estimated sample size of 711. We then calculated a 10% non-response rate. Thus, bringing the estimated sample size to 790. This estimated sample size was then run-up to 800.

## Study variables

### Outcome variable

To assess the burden of dementia, RUDAS which is a widely used screening test for dementia was used. The RUDAS is an easy-to-use instrument with six (6) components that examine memory, body orientation, visuospatial praxis, motor praxis, judgment, and language [10]. The maximum score is 30, with a recommended cut-off score of 23. In other words, “any score of 22 or less should be considered as possible cognitive impairment and referred on for further investigation by the relevant physician”. Higher score indicated superior performance, whereas lower scores indicated poorer cognitive ability or dementia risk. Though not yet validated in Ghana, the RUDAS is a culturally neutral tool that has been used worldwide in Asia, Europe, and Africa [11]. Hence, its usage within the Ghanaian context was acceptable. The psychometric properties of RUDAS have remained moderate to strong over time and across many cultures [12, 13]. For this study, calculated Cronbach’s alpha estimating RUDAS’ reliability was an acceptable value of 0.71, according to conventional cut-offs [14].

### Associated factors

The study analysed age, marital status, education, employment, financial situation, living status (household size), health insurance, and household wealth index as associated factors of dementia, which is consistent with established literature showing that these factors significantly predict dementia [15–17]. These variables were measured with a binary or multi-categorical response scale except for living status (household size) which was measured on a continuous scale asking participants to provide the number of people living in their households. Table 1 contains the list of all explanatory variables with their response options. The household wealth of participants was created using household characteristics, possessions, and assets (e.g., improved water, improved toilet, clean fuel, mobile device, television, fridge/freezer, radio, gas cooker, bicycle, sofa/sofa set, video recorder/DVD player). These assets were originally measured with a mixture of response scales including binary, categorical and continuous response scales. But for ease of creating the index using principal component analysis, all the response scales were recoded into a binary response [18]. Household wealth was categorized into poorest (0), second quintile (1), middle (2), fourth quintile (3), and richest (4).

**Table 1:** Summary characteristics of study variables in the total and male-female stratified samples

	Total sample (*N* = 800)	Male sample (*n* = 291)	Female sample (*n* = 509)
Variables	Frequency (%)	Frequency (%)	Frequency (%)
**Age (in years)**			
45–54	318 (39.75)	99 (34.02)	219 (43.03)
55–64	261 (32.63)	107 (36.77)	154 (30.26)
65–74	161 (20.13)	60 (20.62)	101 (19.84)
75+	60 (7.50)	25 (8.59)	35 (6.88)
**Marital Status**			
Single	48 (6.00)	15 (5.15)	33 (6.48)
Married	492 (61.50)	213 (73.20)	279 (54.81)
Divorced	87 (10.88)	34 (11.68)	53 (10.41)
Separated	29 (3.63)	7 (2.41)	22 (4.32)
Widowed	144 (18.00)	22 (7.56)	122 (23.97)
**Gender**			
Female	509 (63.63)		
Male	291 (36.38)		
**Education**			
No formal education	119 (14.88)	23 (7.90)	96 (18.86)
Graduated from Primary	177 (22.13)	48 (16.49)	129 (25.34)
Graduated from JSS/JHS	327 (40.88)	119 (40.89)	208 (40.86)
Graduated from SSS/SHS	126 (15.75)	68 (23.37)	58 (11.39)
Graduated from Tertiary	51 (6.38)	33 (11.34)	18 (3.54)
**Employment**			
No	289 (36.13)	102 (35.05)	187 (36.74)
Yes	511 (63.88)	189 (64.95)	322 (63.26)
**Financial Status**			
Better	78 (9.75)	27 (9.28)	51 (10.02)
About the same	194 (24.25)	78 (26.80)	116 (22.79)
Worse off	528 (66.00)	186 (63.92)	342 (67.19)
**Living status** (household size)	*M* = 4.10 (*SD =* 2.70)	*M* = 4.12 (*SD =* 2.68)	*M* = 4.01 (*SD =* 2.72)
**Health Insurance**			
No	75 (9.38)	45 (15.46)	30 (5.89)
Yes	725 (90.63)	246 (84.54)	479 (94.11)
**Household wealth index**			
Poorest	165 (20.65)	49 (16.84)	116 (22.83)
Poorer	155 (19.40)	51 (17.53)	104 (20.47)
Middle	166 (20.78)	65 (22.34)	101 (19.88)
Rich	216 (27.03)	86 (29.55)	130 (25.59)
Richest	97 (12.14)	40 (13.75)	57 (11.22)

*Note*. M = Mean; SD = Standard deviation; JSS = Junior Secondary School; JHS = Junior High School; SSS = Senior Secondary School; SHS = Senior High School

### Data collection procedure

Prior to the data collection, ten research assistants were recruited. These research assistants were registered nurses who had recently graduated, pending their placement. The research assistants participated in a two-day intensive training that sought to brief them about the purpose of the study, the procedures, and build their capacity to collect quality data. There was pre-testing of the questionnaire at the University Hospital, KNUST to ensure that it is accurate and clearly understood. Some modifications were made to the initial questionnaire. Once these modifications had been finalised, the research assistants set off to conduct the actual data collection. The data collection exercise lasted from April 18 until May 3, 2023. This was done at premises of the eight healthcare facilities. We targeted only general out-patient department (OPD) attendants, except for Chesire home which does not have an OPD and data collection had to be conducted at the visiting lounges of the facility. All participants were briefed about the study, the duration and their rights to participate or withdraw. Participants were only included if they had given an oral or written informed consent. All participants were evaluated using the standardized Rowland Universal Dementia Assessment Scale (RUDAS) instrument. On average, it took about 10 min for an assessor to administer the test to a participant.

### Statistical analyses

Statistical analyses were performed in Stata version 14 (StataCorp, College Station, TX, USA). Prior to the analysis, data cleaning which involved establishing an analytical sample and recoding variables was completed. The purpose of this step was to ensure that the variables of interest were categorized accordingly for the purposes of logistic regression. Descriptive statistics such as mean, standard deviation, frequencies, and percentages, univariate analyses were computed. Prevalence of dementia for the total sample, and male-female stratified sample with their associated confidence intervals were calculated. A series of logistic regression models were performed with dementia as outcome variable and nine explanatory variables. The measured independent variables used were age, gender, marital status, education, employment, financial situation, living status (household size), health insurance, and household wealth index, all of which have been consistently found in literature to be established factors predicting dementia or cognition in Ghana and beyond [15–17]. The logistic regression analysis was first run on the total sample and repeated on the male and female samples respectively. Adjusted odds ratios, confidence intervals, and p-values were reported for the total sample model, male and female sample models (see Table 3). Missing data was not a major concern affecting the analysis given that all the variables except for wealth status (with 0.001 missing) had zero missing response. Therefore, the default missing data approach, excluding cases listwise which retained cases with only full data was used, hence data of only 799 cases was used in the final analysis.

### Ethical considerations

The Ghana Health Service Ethics Review Committee (GHS-ERC) [ID Number: GHS-ERC: 005/02/23] and the School Research Ethics and Integrity Committee (SREIC), University of Huddersfield, United Kingdom (SREIC Reference: SREIC_ExtApp_2023_001) approved the conduct of this study. Also, permissions were obtained from Kumasi, Ejisu, and Juaben Metropolitan/Municipal Health Directorates where the sampled healthcare facilities were situated. Furthermore, we obtained permission from all the healthcare facilities where the study participants were recruited.

Potential participants were approached before the survey for their informed consents, after they were given a thorough description of the aim, benefits, and risks of the study. It was made clear that participation in the study was entirely voluntary and that participants had the right to decline to take part or withdraw at any time during the data collection. Also, they could ask to withdraw their data only up to 2 weeks after the data collection.

## Results

### Summary of participants’ characteristics

The majority of the participants were females (63.63%), aged 45 to 54 years (39.75%), had junior high or secondary school (JSS/JHS) education as highest qualification (40.88%), currently married (61.50%), and employed (63.88%). More than half (66.00%) perceived their financial status to be worse off than others. Majority of the participants had health insurance (90.63%) and belonged to high (rich) household wealth index (27.03%). Male and female variations were found to have similar distributions. In terms of percentages, there were more female participants aged 45–54 years (43.03%), more married men (73.20%), more JHS graduates (40.89%), and more employed men (64.95%). However, a higher percentage of women (67.19%) thought their financial situation was worse. More women (94.11%) had health insurance. More males indicated belonging to rich households (29.55%). For the total sample, 4.10 and 2.70 were observed to be the respective mean and standard deviation for living status (household size). Table 1 contains more details on the summary statistics.

### Prevalence of dementia

The overall prevalence of dementia risk was 23.38% [95% CI:20.44, 26.31]. More females 24.56% [95% CI:20.81, 28.31] compared to males 21.31% [95% CI:16.57, 26.04] were at risk of dementia (see Table 2).

**Table 2:** Prevalence of dementia for the total, male and female samples

Samples	Prevalence
Total	23.38% [95% CI:20.44, 26.31]
Male	21.31% [95% CI:16.57, 26.04]
Female	24.56 [95% CI:20.81, 28.31]

### Logistic models showing the associated factors of dementia

Adjusted odds ratios are reported for both total and male-female stratified samples in Table 3. Age, marital status, gender, education, employment, financial situation, living status (household size), health insurance, and household wealth index were all regressed on dementia. In the total sample model, the only factors strongly linked to dementia were age and education. However, in the male-female stratified sample, the significant factors associated with dementia varied by age, education, and household wealth index.

**Table 3:** Logistic regression models predicting dementia for the total sample and male-female samples

Variables	Total sample		Male sample		Female sample	
	OR [95% CI]	*p*	OR [95% CI]	*P*	OR [95% CI]	*p*
**Age (in years)**						
45–54	0.41 [0.19, 0.86]	0.019	1.35 [0.35, 5.24]	0.665	0.26 [0.10, 0.66]	0.005
55–64	0.46 [0.23, 0.95]	0.036	0.88 [0.25, 3.17]	0.851	0.35 [0.14, 0.87]	0.023
65–74	0.59 [0.29, 1.17]	0.132	0.54 [0.15, 1.90]	0.338	0.63 [0.26, 1.51]	0.302
75+	1		1		1	
**Marital Status**						
Single	1		1		1	
Married	0.75 [0.35, 1.61]	0.467	2.37 [0.42, 13.40]	0.329	0.59 [0.24, 1.44]	0.247
Divorced	0.80 [0.33, 1.93]	0.614	3.24 [0.49, 21.29]	0.221	0.55 [0.19, 1.63]	0.283
Separated	1.07 [0.35, 3.27]	0.900	5.88 [0.53, 65.83]	0.150	0.77 [0.21, 2.83]	0.690
Widowed	1.22 [0.52, 2.83]	0.648	6.11 [0.77, 48.75]	0.088	0.90 [0.34, 2.37]	0.832
**Gender**						
Female	1					
Male	1.27 [0.85, 1.91]	0. 248				
**Education**						
No formal education	1		1		1	
Graduated from Primary	0.55 [0.33, 0.92]	0.023	0.84 [0.27, 2.56]	0.757	0.52 [0.29, 0.96]	0.036
Graduated from JSS/JHS	0.38 [0.23, 0.62]	< 0.001	0.42 [0.14, 1.22]	0.112	0.39 [0.22, 0.70]	0.002
Graduated from SSS/SHS	0.09 [0.04, 0.22]	< 0.001	0.13 [0.03, 0.49]	0.003	0.06 [0.01, 0.28]	< 0.001
Graduated from Tertiary	0.05 [0.01, 0.24]	< 0.001	0.14 [0.02, 0.88]	0.036	- -	-
**Employment status**						
No	1		1		1	
Yes	0.93 [0.61, 1.40]	0.713	0.56 [0.26, 1.19]	0.132	1.16 [0.69, 1.95]	0.585
**Financial Status**						
Better	0.86 [0.44, 1.70]	0.667	1.78 [0.50, 6.39]	0.374	0.66 [0.29, 1.50]	0.319
About the same	1.00 [0.65, 1.53]	0.985	2.01 [0.96, 4.19]	0.063	0.72 [0.41, 1.26]	0.247
Worse off	1		1		1	
**Living status (household size)**	0.97 [0.91, 1.04]	0.406	0.99 [0.87, 1.14]	0.931	0.96 [0.88, 1.04]	0.310
**Health Insurance**						
No	1		1		1	
Yes	0.67 [0.37, 1.21]	0.180	0.72 [0.30, 1.65]	0.417	0.74 [0.30, 1.84]	0.514
**Household wealth index**						
Poorest	1		1		1	
Poorer	0.85 [0.50, 1.42]	0.527	0.43 [0.17, 1.11]	0.082	1.04 [0.54, 1.99]	0.913
Middle	0.89 [0.53, 1.49]	0.650	0.41 [0.16, 1.02]	0.055	1.18 [0.61, 2.27]	0.629
Rich	0.82 [0.48, 1.38]	0.454	0.37 [0.15, 0.94]	0.036	0.98 [0.50, 1.93]	0.961
Richest	0.62 [0.29, 1.31]	0.206	0.09 [0.02, 0.49]	0.005	1.02 [0.42, 2.52]	0.962
**More details**						
Sample	799		291		490	
Pseudo *R*^*2*^	0.12		0.17		0.12	
F statistics	*F* (21, 799) = 103.6, *p* < 0.001		*F* (20, 291) = 52.2, *p* < 0.001		*F* (19, 490) = 66.1, *p* < 0.001	

*Note*. JSS = Junior Secondary School; JHS = Junior High School; SSS = Senior Secondary School; SHS = Senior High School

### Total sample model

A significant negative relationship was observed between age and dementia such that the younger the age, the less likely the risk of dementia. Specifically, respondents aged 45–54 years [AOR = 0.41, 95% CI:0.19, 0.86, *p* = 0.019], and 55–64 years [AOR = 0.46, 95% CI:0.23, 0.95, *p* = 0.036] were less likely to risk having dementia than those aged 75 and above. Moreover, higher educational level was negatively associated with dementia risk. As shown in the results, respondents who reported receiving formal education such as primary [AOR = 0.55,95% CI:0.33, 0.92, *p* = 0.023], JHS/JSS [AOR = 0.38,95% CI:0.23, 0.62, *p* = 0.001], SHS/SSS [AOR = 0.09,95% CI:0.04, 0.22, *p* = 0.001], and tertiary [AOR = 0.05, 95% CI: 0.01, 0.24, *p* = 0.001] were less likely to report dementia risk than those who had no formal education.

### Male sample model

Educational level in the male sample model was significantly associated with the risk of dementia, such that male respondents who had any form of formal education are less likely to be at risk of dementia compared to their counterparts who had no formal education. Specifically, male respondents who graduated from SHS/SSS [AOR = 0.13, 95% CI:0.03, 0.49, *p* = 0.003] and tertiary [AOR = 0.14, 95% CI:0.02, 0.88, *p* = 0.036] are less likely to be at risk of dementia compared to those who had no form of formal education. Also, household wealth index was negatively related to dementia risk, such that the higher the household wealth index, the lesser chances of being at risk of dementia. Specifically, male respondents belonging to poorest households were more likely to be at risk of dementia compared to those belonging to rich households [AOR = 0.37, 95% CI:0.15, 0.94, *p* = 0.036] and richest households [AOR = 0.09, 95% CI:0.02, 0.49, *p* = 0.005].

### Female sample model

Age in the female sample model was significantly negatively related to dementia risk, such that the younger the age, the less likelihood of being at risk of dementia. Precisely, female respondents aged 45–54 years [AOR = 0.26, 95% CI:0.10, 0.66, *p* < 0.005] and those aged 55–64 years [AOR = 0.35, 95% CI:0.14, 0.87, *p* < 0.023] are less likely to be at risk of dementia compared to those aged 75 and more. Moreover, increasing education levels were associated with less likelihood of being at risk of dementia. For instance, female respondents who graduated from primary [AOR = 0.52, 95% CI:0.29, 0.96, *p* < 0.036], JHS/JSS [AOR = 0.39, 95% CI:0.22, 0.70, *p* < 0.002], and SHS/SSS [AOR = 0.06, 95% CI:0.01, 0.28, *p* = 0.001] were less likely to be at risk of dementia compared to those who had no form of formal education.

## Discussion

This study sought to assess the gendered differences in the prevalence and associated factors of dementia in the Ashanti Region of Ghana. Generally, the prevalence of dementia was 23% which is similar to the prevalence rates of 2-21% reported amongst older adults aged 60 + in a previous systematic review of studies from SSA [19]. Our findings of higher prevalence of dementia in females than males also concur with Nichols et al.’s study [2]. Our findings indicate that the significant associated factors of dementia risk were education, wealth status, and age.

Education emerged as a significant predictor of dementia risk in both males and females. According to our findings, having higher educational attainment was associated with lower risk of dementia. This aligns with previous studies that have found an inverse association between the level of educational attainment and dementia risk [20, 21]. The observed association is corroborated by Liu et al.’s study [22] that showed that higher education reduces the risk of dementia by 8–44%. This finding may be explained from the perspective that formal education creates a lifetime opportunity for intellectual enrichment and stimulation. This “lifetime intellectual enrichment may provide an important brain reserve mechanism to delay the onset of cognitive decline and dementia” [23]. Moreover, persons with higher educational attainment are more likely to be exposed to health information about the modifiable risk factors of dementia. This information gained may influence individuals to engage in health-promoting activities including healthy eating, staying physically active, and having regular health checks which are quintessential to mitigating any pathological factors that can trigger dementia. Our finding is, however, inconsistent with Mirza et al.’s study [20] that revealed that the decrease in the risk of dementia with increasing education was only significant in males.

Evidence from this current study suggests that the risk of dementia by age differed significantly for only females. Women in the oldest age (i.e. 75 years and older) category were at a higher risk of dementia compared to those in the ‘younger’ age group. A similar finding has been reported by Shaw et al. [24] whose study indicates that women in the oldest age had 22% higher likelihood of developing dementia compared to their male counterparts. The plausible reason for the gendered differences in the risk of dementia by age, which is espoused by Beam et al. [25], is due to the point that women live longer (have longer life expectancy) compared to men. Consequently, women have a greater chance of reaching the age group where dementia risk is high compared to men. Another perspective to this could be elucidated by examining the multifaceted interplay of societal expectations, deeply ingrained gender roles, and cultural influences. Traditional gender and socio-cultural norms often impact negatively in terms of educational opportunities and career choices for women, influencing cognitive reserve and resilience against neurodegenerative conditions. This historical confinement of women to certain roles has the potential to limit access to intellectual challenges and cognitive-stimulating activities, affecting cognitive health in the long term in their old age.

Our study also revealed that there was an inverse association between wealth status and dementia risk. This association was significantly gendered; higher wealth status among males was associated with lower likelihood of dementia. However, this was not significant for females. This outcome holds significance as it reinforces the argument that men exposed to environments marked by extensive wealth inequality are at a greater risk of experiencing increased instances of neurodegenerative disorders, including dementia [26, 27]. Again, our finding that there is an inverse association between wealth status and dementia risk is consistent with the findings of a population-based study [28]. A possible explanation for this is that individuals in higher wealth status are more likely to be health literate [29] and have unrestrictive access to health care [30]. This places at an advantageous position of gaining information on how to reduce one’s susceptibility to dementia which individuals in lower wealth statuses may not have. It is, therefore, not surprising that the association between wealth status and dementia risk was gendered in favour of males. The Ghanaian sociocultural system ascribes the role of money-making and wealth accumulation as a male gender role in most instances. Hence, men in poorer wealth status are often ridiculed and ostracised by the society. This could lead to chronic depression – a known risk factor of dementia [31, 32].

### Policy implications

The policy implication of developing gender-specific dementia prevention strategies is rooted in the recognition that gender plays a significant role in determining an individual’s risk of developing dementia. To effectively address and reduce the risk of dementia in both men and women, it is crucial to tailor prevention efforts to the risk factors specific to each gender. The findings underscore a need for Ghana to strengthen formal education for all people, irrespective of being male or female. There is a need to invest in adult education and lifelong learning programmes to ensure that people of all ages can continue to engage in intellectually stimulating activities. For males, interventions aimed at reducing dementia risk must target those in lower wealth status. Women in the oldest age group should be the priority focus for programmes aimed at reducing dementia risk among women. Implementing these policy recommendations would definitely come with some challenges, particularly due to the variability in healthcare infrastructure and accessibility across different regions of Ghana. Rural areas may face limited resources and healthcare facilities, making it challenging to implement targeted dementia prevention interventions.

### Strength and limitations

This study is arguably the first of its kind in Ghana to assess the gendered differences in the prevalence and associated factors of dementia. Thus, contributing to advancing the current knowledge on dementia risk in SSA. Also, using the RUDAS assessment tool provided us with a quick way of screening for dementia risk among the population. Notwithstanding, there are some limitations. The use of the RUDAS assessment tool does not provide us with insights into the specific dementia sub-typologies. Additionally, given that we recruited only persons who visited the healthcare facilities, the findings may not be generalizable to persons with dementia (cognitive impairments) aged 45 years and above who did not visit the healthcare facilities. Also, since the study was cross-sectional in nature, we are unable to infer any sort of causality in the predictors of dementia. Future studies can consider implementing longitudinal studies to be able to establish causal pathways between the factors associated with dementia risk among males and females.

## Conclusion

Based on the findings of the study, we conclude that the prevalence of dementia risk is high, especially among females compared to their male counterparts. Also, there are gendered differences in the associated factors of dementia in Ghana. While there are no differences in terms of the association between educational attainment and dementia risk, there are existing differences for wealth status and age for males and females, respectively. It is, therefore, imperative for Ghana’s Ministry of Health, Ministry of Education, and the Ghana Health Service to consider collaborating on allocating resources towards adult education and lifelong learning initiatives to enable individuals of all age groups to participate in intellectually enriching pursuits. Specifically, when addressing dementia risk in males, interventions should be directed towards those with lower wealth status. Likewise, when developing programmes to mitigate dementia risk in women, particular attention should be given to women in the oldest age category.

### Electronic supplementary material

Below is the link to the electronic supplementary material.

Supplementary Material 1

## Data Availability

All relevant data are within the paper. Any other data or material associated with this manuscript are available on request through the first author (PAD).

## Abbreviations

CI: Confidence interval
JHS: Junior High School
JSS: Junior Secondary School
KNUST: Kwame Nkrumah University of Science and Technology
LMICs: Low-and-middle-income countries
OPD: Out-patient departments
OR: Odds ratio
RUDAS: Rowland Universal Dementia Assessment Scale
SSA: Sub-Saharan Africa
SHS: Senior High School
SSS: Senior Secondary School
