# Supplementary material for: Gendered differences in the prevalence and associated factors of dementia in Ghana: a cross-sectional survey
Source: BMC Psychiatry. 2024 May 27;24:397. doi: 10.1186/s12888-024-05856-3 (PMC11131303; doi:10.1186/s12888-024-05856-3)
Supplement: Supplementary file 1 — Supplementary Material 1 [file 12888_2024_5856_MOESM1_ESM.pdf]

Supplementary table: Capacity of healthcare facilities included in the study

Facilities	Bed capacity	Annual OPD	Annual IPD	Participants sampled
Ejisu government hospital	95	6679	625	112
Chesire home	55	300	22	16
Kumasi South hospital	120	142028	7242	112
Onwe government hospital	58	16685	2640	112
Tafo government hospital	58	793785	9452	112
Juaben government hospital	88	37807	4811	112
Manhyia government hospital	123	153657	11492	112
KNUST hospital	125	93902	11846	112
